# Feasibility of hemispatial neglect rehabilitation with virtual reality-based visual exploration therapy among patients with stroke: randomised controlled trial

**DOI:** 10.3389/fnins.2023.1142663

**Published:** 2023-04-20

**Authors:** Joon-Ho Shin, Mingyu Kim, Ji-Yeong Lee, Mi-Young Kim, Yu-Jin Jeon, Kwanguk Kim

**Affiliations:** ^1^Department of Rehabilitation Medicine, National Rehabilitation Center, Ministry of Health and Welfare, Seoul, Republic of Korea; ^2^Department of Computer Science, Hanyang University, Seoul, Republic of Korea

**Keywords:** hemispatial neglect, stroke, rehabilitation, virtual reality, digital

## Abstract

**Background:**

Hemispatial neglect (HSN) was diagnosed using a virtual reality-based test (FOPR test) that explores the field of perception (FOP) and field of regard (FOR). Here, we developed virtual reality-visual exploration therapy (VR-VET) combining elements from the FOPR test and visual exploration therapy (VET) and examined its efficacy for HSN rehabilitation following stroke.

**Methods:**

Eleven participants were randomly assigned to different groups, training with VR-VET first then waiting without VR-VET training (TW), or vice versa (WT). The TW group completed 20 sessions of a VR-VET program using a head-mounted display followed by 4 weeks of waiting, while the WT group completed the opposite regimen. Clinical HSN measurements [line bisection test (LBT), star cancellation test (SCT), Catherine Bergego Scale (CBS), CBS perceptual-attentional (CBS-PA), and CBS motor-explanatory (CBS-ME)] and FOPR tests [response time (RT), success rate (SR), and head movement (HM) for both FOP and FOR] were assessed by blinded face-to-face assessments.

**Results:**

Five and six participants were allocated to the TW and WT groups, respectively, and no dropout occurred throughout the study. VR-VET considerably improved LBT scores, FOR variables (FOR-RT, FOR-SR), FOP-LEFT variables (FOP-LEFT-RT, FOP-LEFT-SR), and FOR-LEFT variables (FOR-LEFT-RT, FOR-LEFT-SR) compared to waiting without VR-VET. Additionally, VR-VET extensively improved FOP-SR, CBS, and CBS-PA, where waiting failed to make a significant change. The VR-VET made more improvements in the left hemispace than in the right hemispace in FOP-RT, FOP-SR, FOR-RT, and FOR-SR.

**Conclusion:**

The observed improvements in clinical assessments and FOPR tests represent the translatability of these improvements to real-world function and the multi-dimensional effects of VR-VET training.

**Clinical trial registration:**

https://clinicaltrials.gov/ct2/show/NCT03463122, identifier NCT03463122.

## 1. Introduction

Hemispatial neglect (HSN) manifests as slow, incomplete, or a lack of responses to stimuli presented in the area of the visual field contralateral to the injured hemisphere (Heilman et al., 2000; Gillen et al., 2005). Although many patients experience notable recovery in the early phase after injury, the symptoms of HSN can persist for many years. Numerous studies have aimed to advance our understanding of HSN rehabilitation; however, a recent Cochrane Review has suggested that there is insufficient evidence to support the efficacy of available strategies (Longley et al., 2021).

Recent studies have investigated the application of virtual reality (VR) for HSN rehabilitation (Martino Cinnera et al., 2022). Interactions with realistic, computer-generated environments in VR expose users to rich multimodal sensory information. Moreover, VR technology permits the precise control of stimuli, as well as objective and detailed measurements of performance. Several studies have also reported that VR outperforms conventional rehabilitation and increases the ability of patients with HSN to perform activities of daily living (Kim et al., 2011; Fordell et al., 2016). Recently, wearable head-mounted displays (HMDs) have gained popularity, as they provide a more realistic interactive experience, acquire spatial information more accurately, and are affordable (Hendrix and Barfield, 1996; Ruddle et al., 1999). Moreover, HMDs have demonstrated benefits in the area of HSN intervention (Martino Cinnera et al., 2022).

In previous studies, we proposed the FOPR test, a novel HMD-based VR visuospatial function test that screens for HSN using the concept of field of perception (FOP) and field of regard (FOR). FOP refers to the size/angle of the visual field that is visible at any given moment without head and body movement, whereas FOR refers to the total area of visual field obtained with the head or body movement to view the surroundings (Jang et al., 2016; Kim et al., 2021).

Based on our previous findings, we infer that our VR-based visuospatial function test can be applied to HSN rehabilitation, which encompasses FOR training with a visual cue (Jang et al., 2016). This work could be linked to visual exploration therapy (VET), which manipulates the participant’s spatial attention toward the neglected space by employing guidance with auditory or visual cues on the neglected side. Previous studies have demonstrated that VET is more effective in improving symptoms of neglect than usual cognitive training (Antonucci et al., 1995). VET improved oculomotor function and searching (van Wyk et al., 2014). VET also modulates attentional demands in visual search and aids in relearning economical search strategy (Kerkhoff, 1998). Despite the multiple positive reports of VET mentioned, the evidence is still not established (Gammeri et al., 2020; Longley et al., 2021). On the other hand, the effects of VET has been enhanced via combination with other techniques (Gammeri et al., 2020). Similarly, VET can be easily implemented in VR (Tsirlin et al., 2009). VR could overcome the shortcomings of VET, which requires considerable therapist participation, as well as patient co-operation, to attain stable and generalised effects (Antonucci et al., 1995; Kerkhoff and Schenk, 2012). In addition, VR presents intense, multi-sensory interaction, enabling enhancement of attention (Martino Cinnera et al., 2022). Therefore, we hypothesised that our VET-incorporating VR-based rehabilitation, hereinafter referred to as the VR-VET, would improve HSN. Fordell et al. (2016) combined VR and VET to demonstrate the effectiveness of the combination; however, the study was performed using a single-group pretest-posttest design. Hence, it is difficult to distinguish whether the results are due to natural recovery or a specific intervention. A recent study utilized VR and motion tracking sensor and reported the resulting effects on HSN; however, the study only used general, rather than specialized, games for HSN, and the recovery mechanisms therein were not clearly described (Choi et al., 2021).

Therefore, we conducted a feasibility study to assess the effectiveness of VR-VET in managing HSN in patients with stroke. We assessed the effects of VR-VET comparing between “VR-VET training” and “waiting without VR-VET training” using a randomised controlled, cross-over experimental design with traditional HSN assessment, as well as FOPR test outcomes. Additionally, we analyzed and compared the VR-VET effects by dividing the left and right hemispaces.

## 2. Materials and methods

### 2.1. Participants

Twenty participants with left HSN were consecutively recruited from our rehabilitation hospital, and 11 participants meeting the following criteria took part in this study: (1) presence of left HSN as demonstrated by a score above the cut-off, which is 7 on the line bisection test (LBT) or 51 on the star cancellation test (SCT) of the Behavioural Inattention Test (Wilson et al., 1987b), or mild-to-moderate neglect-related functional impairment defined by a score of 1–20 points on the Catherine Bergego Scale (CBS) (Azouvi et al., 2003); (2) aged 19–65 years; (3) first-time right hemispheric ischemic or haemorrhagic stroke as evidenced by brain imaging and medical records; (4) a score of >25 on the Mini-Mental State Examination (Folstein et al., 1975); and (5) ability to click responses with the computer mouse buttons. Exclusion criteria were as follows: (1) presence of oculomotor palsies via standard neurological examination; (2) visual defects (including hemianopia) assessed with confrontation test and Goldman kinetic perimetry performed by an ophthalmologist (Elliott et al., 1997); (3) orthopaedic disorders affecting neck movement; and (4) inability to maintain a seated position.

### 2.2. Study design

**FIGURE 1:**
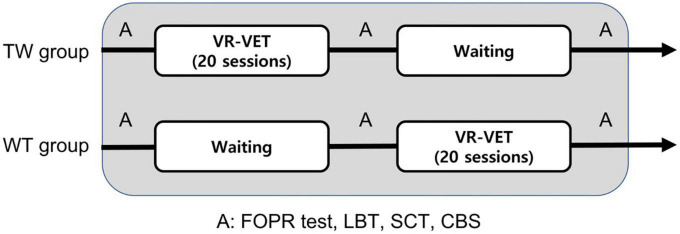
Schematic representation of the rehabilitation program. A, assessment; CBS, Catherine Bergego Scale; LBT, line bisection test; SCT, star cancellation test; TW, training first followed by waiting; WT, waiting first then training; VR-VET, virtual reality-based visual exploration therapy.

The present study was a single-centre, single-blinded, randomised controlled trial with a cross-over design. Eligible participants were randomly assigned to one of two groups [training with VR-VET followed by waiting without VR-VET training [TW] or waiting without VR-VET then training with VR-VET (WT)] using a computer-generated randomisation table. Group allocation was performed using sealed opaque envelopes that had been ordered numerically. The TW group completed 20 sessions of a VR-VET program using an HMD (five daily sessions per week over a period of 4 weeks) followed by 4 weeks of waiting without VR-VET, while the WT group completed the opposite regimen (Figure 1).

The same amount of conventional rehabilitation was given to both VR-VET training and waiting throughout the intervention period. Conventional rehabilitation consisted of usual stroke rehabilitation (physiotherapy and occupational therapy; 30 min of each 5 days per week) and neuropsychological intervention for HSN (sensory or verbal cueing, visual scanning, and movement-based intervention; 30 min of intervention 3 days per week). Thus, the only difference between VR-VET training and waiting was the provision of the VR-VET intervention. Patients completed study assessments before and after the VR-VET training and waiting period by face-to-face method. Each assessment included clinical HSN assessments (LBT, SCT, and CBS) and HMD-provided assessments (FOR and FOP measures; FOPR test), which are further described in the following section. Every assessment was performed by an experienced research occupational therapist, blinded to the group allocation. Interventions and assessments were done in a separate research room in the rehabilitation hospital. Randomisation and outcome measurements were performed by research therapists who were not involved in the study intervention.

This study was registered at clinicaltrials.gov (NCT03463122) and approved by the ethics committee of the institutional review board of National rehabilitation centre, and all procedures/experiments were performed at the rehabilitation hospital in accordance with relevant guidelines and regulations. All participants provided written informed consent prior to study participation.

### 2.3. Apparatus

The VR-VET and FOPR test were created using a three-dimensional development platform (Vizard 4.0; World Viz, Santa Barbara, CA, USA) and implemented using a stereo HMD system (Oculus Rift DK1, Oculus VR, Irvine, CA, USA). The resolution of the HMD screen was 1280 × 800 (640 × 800 per eye); the diagonal FOP was 110° (horizontal range: 93.3°; vertical range: 58.3°); and a built-in, three-degrees-of-freedom sensor was used to track head movements. The system was controlled by a desktop workstation running Windows 7 (Microsoft) and equipped with a high-end graphics card (NVidia GTX 760Ti). A standard computer mouse was used for patient responses.

#### 2.3.1. VR-VET

**FIGURE 2:**
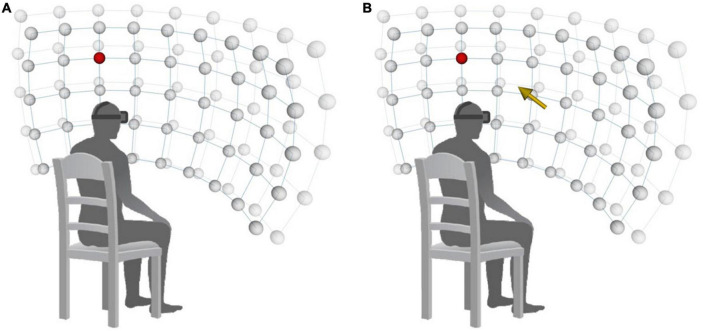
Arrangement of stimuli on a 9 × 5 × 2 spherical coordinate system. **(A)** VR-VET-no cue. **(B)** VR-VET-cue. VR-VET, virtual reality-based visual exploration therapy.

During VR-VET, the head-tracking feature of the HMD was activated so that the participant’s view of the screen changed in accordance with head rotation. Then, the participants were asked to move their heads to detect a target as quickly as possible. The colour of the target was red or blue, with each colour presented in a random order at each target position. Participants were instructed to click the left or right button of a computer mouse when they saw a blue or red target, respectively. There were 90 potential target positions distributed over a 9 × 5 × 2 (horizontal × vertical × radial) spherical coordinate system, and each target was located at interval distances of 15° from the centre of the screen (Figure 2A; horizontal range: 120°; vertical range: 60°; and near–far positions in the radial direction). Each target appears only when the patients fix their head in the midline by aligning the white crosses, which represent the centre of the current visual field, with the red crosses, which represent the centre of virtual space.

Every target was presented once in a randomised order. Participants completed two different blocks of VR-VET in each session, for a total of 180 targets of doses per session, which lasted approximately for 20–25 min. In the first block (VR-VET-no cue), a white fixation cross was displayed in the centre of the screen before each trial. At the beginning of each trial, the fixation cross disappeared, and participants were instructed to find the target as quickly as possible. When participants pressed a button to indicate detection of a target, the target immediately disappeared; if participants failed to detect the target within 10 s, they automatically proceeded to the next trial. Auditory feedback was provided to indicate trial success or failure. After each trial, participants were required to resume the original head position. The inter-trial interval varied between 0.5 and 1.5 s. The second block (VR-VET-cue) was similar to the VR-VET-no cue block, except that a visual cue (i.e., a yellow arrow at the centre of the screen) was provided to indicate the direction of the target stimulus in three-dimensional space at the start of each trial (Figure 2B).

### 2.4. Outcome measures

#### 2.4.1. Clinical HSN assessments

Participants were assessed for symptoms of HSN using the LBT and SCT of the Behavioural Inattention Test (Wilson et al., 1987a). Both tests were performed using an A4 sheet of paper presented in front of the participants’ mid-sagittal line. Higher scores on both tests indicate more severe neglect.

In the LBT, participants were presented with a sheet of paper containing three horizontal lines depicted in a staircase fashion. Participants were asked to bisect the given lines by marking the centre of each line using their preferred or unaffected hand. The deviation of the marking from the true centre of each line was then converted to a score that ranged from 0 to 3, with total scores ranging from 0 to 9.

In the SCT, participants were presented with a sheet of paper containing 56 small stars interspersed among distractors (Wilson et al., 1987b). The experimenter crossed out the two small, central stars of the sheet as an example; following which, participants were asked to mark the remaining small stars. Scores were calculated as the total number of cancelled small stars and ranged from 0 to 54. Additionally, SCT score was used to classify the HSN severity into mild (>43), moderate (38–43), and severe (<38) (Linden et al., 2005).

The CBS is a standardised checklist designed to detect the degree of neglect during everyday life via direct observation of functioning during ten tasks (e.g., grooming, dressing, and wheelchair operation) (Azouvi et al., 2003). Scores on each task range from 0 to 3, resulting in total scores of 0 to 30, with lower scores indicating better function. CBS is subdivided into CBS perceptual-attentional (CBS-PA; six items, ranges from 0 to 18) and CBS motor-explanatory (CBS-ME; four items, ranges from 0 to 12) (Goedert et al., 2012).

#### 2.4.2. HMD-provided assessments (FOPR tests)

Dependent variables for the HMD-provided assessment (FOPR tests) included response time (RT), success rate (SR), and head movement (HM). RT was defined as the time interval between target onset and response(s). SR was defined as the percentage of correct responses (clicking the left or right mouse button in response to a blue or red target, respectively). HM was defined as the sum of all quantified head-rotations. We sampled current frame values of head rotations (yaw, pitch, and roll) at 60 Hz and subtracted the result from the previous frame values ($\Sigma \,\sqrt{△\,y\,a\,w^{2}+△\,p\,i\,t\,c\,h^{2}+△\,r\,o\,l\,l^{2}}$) to yield a final value (°).

FOP measures were included to assess the ability of participants to detect the target in the absence of head movement. For FOP measurements, the head-tracking feature of the HMD was turned off to ensure the participant’s view of the screen remained constant despite participant head movement. Thirty targets were randomly distributed over a 5 × 3 × 2 spherical coordinate system (horizontal range: 60°, vertical range: 30°; and near–far positions in the radial direction) within the FOP of the experimental equipment (Oculus; horizontal: 93.3°; vertical: 58.3°). The dependent variables for FOP measurement included RT (FOP-RT) and SR (FOP-SR).

FOR measures assess the ability of participants to detect the target with the head movement. The head-tracking feature of the HMD was turned on so that the participant’s view of the screen changed as the head moves. That is, FOR measurements were obtained in a manner similar to that used during the VR-VET-no cue trials with the only difference being the target appearance order. Ninety targets were randomly distributed over a 9 × 5 × 2 spherical coordinate system (horizontal range: 120°, vertical range: 60°; and near–far positions in the radial direction). The dependent variables for assessments included RT (FOR-RT), SR (FOR-SR), and HM (FOR-HM), which were calculated separately for the left and right hemispaces.

### 2.5. Statistical analysis

Spearman correlations were computed to assess the linear relationship between HMD variables from near plane and those from far plane. There were positive correlations between whole HMD variables from near plane and far plane (*r* > 0.9, *p* < 0.001). As HSN has been tested and reported in two dimensions (vertical and horizontal), rather than radial direction, we reported the data combining two radial planes with an analytic base, supported by a high correlation between the two planes. A separate analysis from each plane was reported in the Supplementary material.

We performed the Mann–Whitney U-tests and Fisher’s exact tests for continuous and categorical variables, respectively, to compare baseline characteristics between TW and WT groups. To examine the potential effects of training order associated with the crossover design, we compared the change of outcome variables throughout the intervention (either TW or WT) between the TW and WT groups using the Mann–Whitney U-test.

We then conducted repeated measures analyses of variance (RM ANOVA) for clinical HSN (LBT, SCT, and CBS) and FOPR tests: FOP variables (FOP-RT and FOP-SR), FOR variables (FOR-RT, FOR-SR, and FOR-HM), with *Type* (VR-VET vs. waiting) as a between-subjects factor and *Time* (pre vs. post) as a within-subject factor. Before applying RM ANOVA, we examined assumptions of normality and variance homogeneity using the Kolmogorov-Smirnov test and Levene’s *F*-tests, respectively. These assumptions were met, and Greenhouse–Geisser corrections were applied when the violation of sphericity occurred. In addition, paired Wilcoxon tests were conducted on clinical HSN tests (LBT, SCT, and CBS) to compare pre- and post-training values.

Additionally, we analyzed FOPR tests measures for each hemispace with VR-VET. Because all participants exhibited left HSN and underwent identical training for the left and right spaces, responses from the right hemispace were regarded as control values. Among the 90 target stimuli, 80 stimuli were used for right or left hemispace analyses (i.e., 40 left and 40 right), while 10 stimuli on the central line were excluded. We conducted a 2 × 2 ANOVA (pre/post-VR-VET and left/right hemispace) on FOPR tests: FOP variables for the 24 included targets, FOR variables for the 80 included targets.

All statistical analyses were performed using R 4.1.2.^1^ Data are presented as the mean ± standard deviation. *P*-values <0.05 were considered statistically significant.

## 3. Results

### 3.1. Participant characteristics

Eleven right-handed patients diagnosed with left HSN following right hemispheric stroke participated in the present study based on the inclusion and exclusion criteria (age: 54.9 ± 5.2 years; time of intervention from stroke onset: 12.0 ± 15.5 months; CBS score: 6.5 ± 4.8). Five and six participants were allocated to the TW and WT groups, respectively, without any dropout after enrolment (Table 1). At baseline, there were no significant between-group differences in sex (*p* = 0.900), age (*p* = 0.068), education (*p* = 0.328), duration of stroke (*p* = 1.000), LBT score (*p* = 0.540), SCT score (*p* = 0.899), CBS score (*p* = 0.097), or HSN severity (*p* = 0.272). No adverse events were observed during the study.

**TABLE 1:** Participant characteristics.

No.	Group	Sex	Age	Handedness	Education	Storke type	Months from stroke	LBT	SCT	CBS	Severity
1	TW	Male	59	Right	12	Ischaemia	44	0	53	10	Mild
2	TW	Male	60	Right	12	Haemorrhage	5	6	43	11	Moderate
3	WT	Male	60	Right	20	Haemorrhage	41	4	39	3	Mode
4	WT	Male	48	Right	16	Ischaemia	3	9	54	12	Mild
5	TW	Male	62	Right	16	Ischaemia	4	9	52	1	Mild
6	WT	Male	50	Right	16	Haemorrhage	2	0	34	3	Severe
7	TW	Male	57	Right	16	Ischaemia	2	4	39	12	Moderate
8	WT	Male	53	Right	16	Ischaemia	15	8	44	3	Mild
9	WT	Male	47	Right	16	Haemorrhage	7	7	53	2	Mild
10	WT	Female	56	Right	9	Ischaemia	4	5	52	6	Mild
11	TW	Female	52	Right	12	Ischaemia	5	2	40	17	Moderate

CBS, Catherine Bergego Scale; LBT, line bisection test; SCT, star cancellation test; TW, VR-VET training first, then waiting; WT, waiting first, then VR-VET training.

### 3.2. Effect of training order

We observed no significant differences in changes of all FOPR tests between the TW and WT groups: FOP-RT (*p* = 0.121), FOP-SR (*p* = 0.08), FOR-RT (*p* = 0.850), FOR-SR (*p* = 0.450), or FOR-HM (*p* = 0.229). Similarly, there were no significant between-group differences in LBT (*p* = 0.560), SCT (*p* = 0.348), or CBS scores (*p* = 0.241) changes. These results indicated no effects of training order, thus enabling further comparisons of VR-VET against waiting.

### 3.3. Effects of VR-VET on clinical HSN measurements

We observed a significant interaction effect of *Type* × *Time* on LBT score (*F* = 5.76, *p* = 0.037), although there were not significant interaction effects of *Type* × *Time* on SCT (*F* = 0.353, *p* = 0.565), CBS score (*F* = 0.641, *p* = 0.442), CBS-PA (*F* = 0.454, *p* = 0.516), and CBS-ME (*F* = 0.361, *p* = 0.561) (Table 2).

**TABLE 2:** Effects of VR-VET and WT on clinical HSN assessments and FOPR tests.

	VR-VET (*n* = 11)		WT (*n* = 11)		Interaction		Main effect: time		Main effect: type	
	**Before**	**After**	**Before**	**After**	***F***	***P***	***F***	***P***	***F***	***P***
LBT	4.4 ± 2.8	6.3 ± 2.0	6.1 ± 2.7	5.3 ± 2.5	5.760	0.037	1.213	0.297	0.505	0.493
SCT	47.4 ± 6.0	49.8 ± 5.4	47.0 ± 7.2	48.2 ± 7.3	0.353	0.565	2.285	0.162	0.596	0.458
CBS	6.5 ± 4.8	4.8 ± 3.8	5.9 ± 4.7	5.5 ± 5.0	0.641	0.442	2.500	0.145	0.017	0.900
CBS-PA	3.5 ± 2.8	2.5 ± 2.2	3.3 ± 2.6	3.1 ± 3.2	0.454	0.516	2.474	0.147	0.225	0.645
CBS-ME	3.0 ± 2.5	2.3 ± 1.8	2.6 ± 2.2	2.5 ± 2.2	0.361	0.561	2.024	0.185	0.068	0.800
FOP-RT (s)	2.0 ± 0.7	1.5 ± 0.7	1.9 ± 0.8	1.6 ± 0.7	2.991	0.114	16.056	0.002	0.023	0.881
FOP-LEFT-RT (s)	3.3 ± 1.2	2.4 ± 1.3	2.9 ± 1.2	2.6 ± 1.5	13.731	0.004	14.180	0.004	0.264	0.619
FOP-RIGHT-RT (s)	1.1 ± 0.6	0.9 ± 0.3	1.3 ± 0.7	0.8 ± 0.2	1.265	0.287	6.734	0.027	0.106	0.751
FOP-SR (%)	73.0 ± 17.0	85.5 ± 14.5	77.4 ± 21.2	82.0 ± 16.6	2.749	0.128	16.207	0.002	0.018	0.897
FOP-LEFT-SR (%)	47.3 ± 31.3	68.6 ± 28.7	59.5 ± 30.6	61.4 ± 32.8	7.664	0.020	8.079	0.017	0.207	0.659
FOP-RIGHT-SR (%)	92.0 ± 11.6	97.0 ± 5.6	89.0 ± 17.4	97.3 ± 3.9	0.341	0.572	7.344	0.022	0.172	0.687
FOR-RT (s)	5.0 ± 1.4	3.0 ± 1.1	4.2 ± 1.6	4.2 ± 1.4	14.334	0.004	32.935	0.000	0.246	0.631
FOR-LEFT-RT (s)	7.1 ± 1.7	4.0 ± 1.9	5.4 ± 2.3	5.8 ± 2.3	21.512	0.001	45.202	0.000	0.055	0.819
FOR-RIGHT-RT (s)	3.4 ± 1.8	2.5 ± 0.8	3.5 ± 1.8	2.9 ± 1.0	0.241	0.634	6.464	0.029	0.553	0.474
FOR-SR (%)	69.1 ± 16.1	91.3 ± 10.1	78.3 ± 18.6	79.7 ± 16.6	12.348	0.006	33.005	0.000	0.081	0.781
FOR-LEFT-SR (%)	45.9 ± 22.8	85.2 ± 20.0	67.5 ± 29.4	62.7 ± 31.7	24.783	0.001	49.815	0.000	0.006	0.941
FOR-RIGHT-SR (%)	87.7 ± 19.7	95.7 ± 3.4	85.7 ± 19.1	93.9 ± 6.4	0.001	0.979	6.466	0.029	0.230	0.642
FOR-HM (°)	8280.5 ± 2551.4	7051.0 ± 3155.0	11550.8 ± 10500.5	9649.3 ± 6276.5	0.026	0.875	1.000	0.341	4.692	0.056
FOR-LEFT-HM (°)	3742.1 ± 2292.9	3396.9 ± 1765.2	3792.8 ± 3450.0	4292.2 ± 5913.8	0.156	0.701	0.006	0.940	0.215	0.653
FOR-RIGHT-HM (°)	4531.9 ± 2468.8	3880.1 ± 1739.5	7479.9 ± 9609.6	5212.8 ± 2464.6	0.263	0.619	1.417	0.261	2.032	0.185

Data are presented as the mean ± standard deviation. VR-VET, virtual reality-visual exploration therapy; FOP, field of perception; FOR, field of regard; RT, response time; SR, success rate; HM, head movement; LBT, line bisection test; SCT, star cancellation test; CBS, Catherine Bergego Scale.

Analyses for LBT score (*p* = 0.058) and SCT (*p* = 0.078) score did not show a significant difference between the pre-VR-VET and post-VR-VET as well as between the pre-waiting and post-waiting (LBT, *p* = 0.254; SCT, *p* = 0.720). Analyses on CBS score revealed a significant difference between the pre-VR-VET and post-VR-VET (*p* = 0.041), but not between the pre-waiting and post-waiting (*p* = 0.676). Similarly, CBS-PA score showed a significant difference between the pre-VR-VET and post-VR-VET (*p* = 0.034) but not between the pre-waiting and post-waiting (*p* = 0.932). On the other hand, CBS-ME did not show significant difference neither for VR-VET (*p* = 0.305) nor for waiting (*p* = 1.000).

### 3.4. Effects of VR-VET on FOPR tests

**FIGURE 3:**
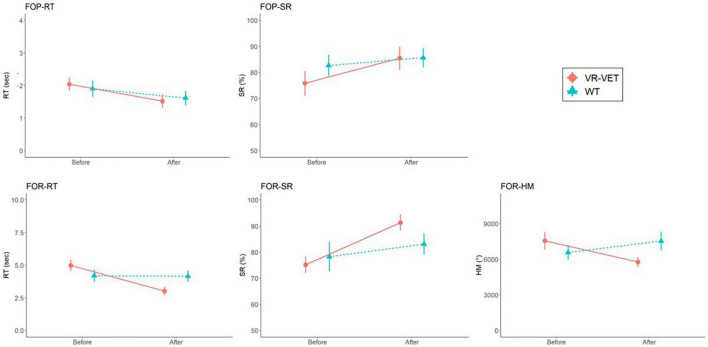
Comparisons of FOPR test in the whole space between VR-VET and waiting. FOP, field of perception; FOR, field of regard; RT, response time; SR, success rate; HM, head movement; VR-VET, virtual reality-visual exploration therapy.

We observed significant interaction effects of *Type* × *Time* on FOR-RT (*F* = 14.334, *p* = 0.004) and FOR-SR (*F* = 12.348, *p* = 0.006) but not on FOP-RT (*F* = 2.991, *p* = 0.114), FOP-SR (*F* = 2.749, *p* = 0.128), and FOR-HM (*F* = 0.026, *p* = 0.875) (Figure 3 and Table 2). *Post hoc* analysis demonstrated significant improvement of FOP-RT throughout both VR-VET (*p* = 0.001) and waiting (*p* = 0.049). Similarly, FOP-SR improved significantly with VR-VET (*p* = 0.005) but not with waiting (*p* = 0.25). HM did not show any significant change with VR-VET (*p* = 0.138) or waiting (*p* = 0.609).

**FIGURE 4:**
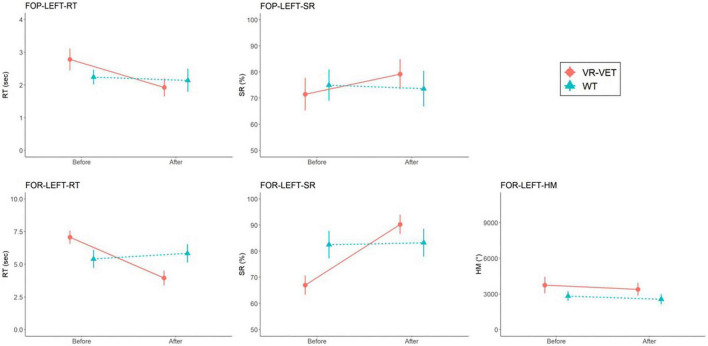
Comparisons of FOPR test in the left hemispace between VR-VET and waiting. FOP, field of perception; FOR, field of regard; RT, response time; SR, success rate; HM, head movement; VR-VET, virtual reality-visual exploration therapy.

For the left FOPR measures, we identified significant interaction effects of *Type* × *Time* on FOP-LEFT-RT (*F* = 13.731, *p* = 0.004), FOP-LEFT-SR (*F* = 7.664, *p* = 0.02), FOR-LEFT-RT (*F* = 21.512, *p* < 0.001), and FOR-LEFT-SR (*F* = 24.783, *p* < 0.001), although there was no significant interaction effect of *Type* × *Time* on FOR-LEFT-HM (*F* = 0.156, *p* = 0.701) (Figure 4 and Table 2). *Post hoc* analysis for FOR-LEFT-HM did not change from either VR-VET (*p* = 0.579) or waiting (*p* = 0.806). There were no interactions on the right FOPR measures. The results of both near and far space are represented in the Supplementary Figures 1–4 and Supplementary Table.

### 3.5. Training effects for each hemispace

**FIGURE 5:**
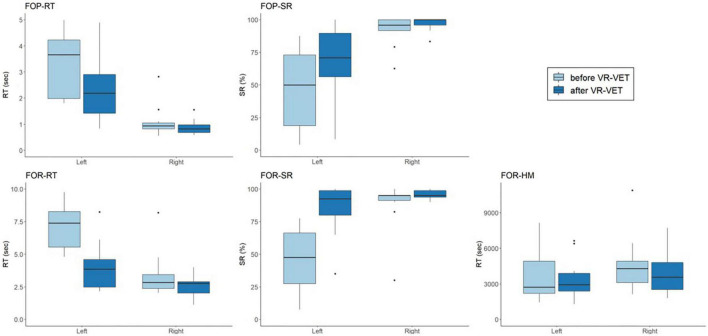
Comparisons of VR-VET effects on FOPR test between the right and left hemispaces. FOP, field of perception; FOR, field of regard; RT, response time; SR, success rate; HM, head movement; VR-VET, virtual reality-visual exploration therapy.

Figure 5 demonstrates the VR-VET effects on FOPR tests on each right and left hemispace. We observed significant interaction effects of *Hemispace* × *Time* on FOP-RT (*F* = 19.169, *p* = 0.001), with significant effects of Time (*F* = 22.409, *p* < 0.001) and Hemispace (*F* = 24.967, *p* < 0.001). Similarly, we identified significant interaction effects of *Hemispace* × *Time* on FOP-SR (*F* = 11.139, *p* < 0.001), with significant main effects of Time (*F* = 22.758, *p* < 0.001) and Hemispace (*F* = 17.778, *p* = 0.002). We observed significant interaction effects of *Hemispace* × *Time* on FOR-RT (*F* = 16.831, *p* = 0.002), with significant effects of Time (*F* = 31.622, *p* < 0.001) and Hemispace (*F* = 29.984, *p* < 0.001). Similarly, we identified significant interaction effects of *Hemispace* × *Time* on FOR-SR (*F* = 23.889, *p* < 0.001), with significant main effects of Time (*F* = 26.380, *p* < 0.001) and Hemispace (*F* = 19.338, *p* = 0.001). However, we did not find significant interaction effects of *Hemispace* × *Time* on FOR-HM (*F* = 0.109, *p* = 0.748). We also did not find main effects of Time (*F* = 1.403, *p* = 0.264) or Hemispace (*F* = 0.831, *p* = 0.383) on FOR-HM.

## 4. Discussion

The present study demonstrated the efficacy of HMD-based VR-VET for the rehabilitation of HSN following stroke. Notably, VR-VET extensively improved more on LBT, FOR variables (FOR-RT and FOR-SR), FOP-LEFT variables (FOP-LEFT-RT and FOP-LEFT-SR), and FOR-LEFT variables (FOR-LEFT-RT and FOR-LEFT-SR) compared to waiting; these effects are from VR-VET effects and not from spontaneous recovery. Additionally, VR-VET considerably improved FOP-SR, CBS, CBS-PA, and FOP-SR, where waiting failed to provoke significant changes. Therefore, we can assert that the VR-VET has practical significance to provide therapeutic effects that generalise to various outcomes.

The improvement with VR-VET could be viewed in many aspects. First, the improvement was not limited to the virtual environment, but generalised to the real world, as demonstrated in the LBT and CBS, which represent the body function/structure and activity, respectively, in terms of International Classification of Functioning, Disability and Health (World Health Organization, 2001). Additionally, the CBS was known to assess personal space, near and far extra-personal spaces, and aspects associated with the perceptual, representational, and motor domains (Menon and Korner-Bitensky, 2004). Taken together, improvements in these clinical tests represent both the translatability of VR-VET improvements to real-world function and VR-VET’s multi-dimensional effects. Improvements may, therefore, be attributable to the acquisition of spatial knowledge in a virtual environment that mimics the physical environment (Ruddle et al., 1999). In line with our results, previous studies reported that VR training on HSN had effects on clinical assessments, such as SCT and CBS (Kim et al., 2011; Fordell et al., 2016). However, these effects have not been consistently reported; thus, the components of the VR system, such as the mode of action, should be considered for the development of VR for HSN (Ogourtsova et al., 2017).

Second, both FOP and FOR measures markedly improved, suggesting the beneficial effects on perceptual-attentional and motor-intentional and exploratory function (Goedert et al., 2014; Kim et al., 2021). The improvement of FOR is easily understood as an acquisition of exploratory strategy followed by integration of perception and exploration, facilitated by repetitive exploratory training in VR-VET. Interestingly, there was no significant change in HM between pre- and post-VR-VET, suggesting that the beneficial effects of VR-VET are caused by the improvement of exploratory quality, not by just increased quantity of exploration. This might indicate that visual stimuli improved saccades or oculomotor function, ultimately resulting in HSN improvement (Kinchla and Wolfe, 1979; van Wyk et al., 2014). On the other hand, it might be related to improved perceptual performance, such as spatial information processing, global attention, speed, or detective performance. This interpretation is supported by the improvement of FOP, which was not trained in the VR-VET. Moreover, the enhancement shown in the CBS-PA, not CBS-ME, also strengthens this interpretation of the beneficial effects of VR-VET, especially on perception.

In summary, VR-VET primarily enhances perceptual performance, and in turn improves exploratory performance not the amount of exploration. This VR-VET’s enhancement of both motor exploratory and perceptual performance is distinct from conventional treatment; the monocular patching and prism adaptation improved perceptual performance and motor-intentional performance, respectively (Barrett and Burkholder, 2006; Fortis et al., 2011). Additionally, both speed (RT) and accuracy (SR) of FOP and FOR measures were significantly improved by our intervention, indicating that the benefits of VR-VET were not subject to trade-offs between speed and accuracy, but to fundamental HSN improvements.

Third, VR-VET effects on FOP-RT & SR and FOR-RT & SR were more prominent in the left hemispace compared to the right hemispace, although the HM change through VR-VET did not differ between the right and left hemispaces. These results suggest that patients experienced asymmetric improvements in perception and exploratory performance toward the left hemispace. We presented the visual targets equally in the right and left hemispace, without any preference on the left hemispace, differently from original VR-VET, which has been mainly employed with leftward cue and left stimuli. Therefore, the effects of VR-VET could be obtained with an equalising process leading to bias reduction to the left space, rather than simple response to stimuli.

An important strength of our VR-VET involved the combination of top-down and bottom-up approaches. Generally, visual exploration therapy can be regarded as a top-down approach that enhances visual scanning or searching, requiring a combination of instruction to attend to the neglected side and participant recognition of neglect. In addition, our intervention contains a VR-VET-cue, where the use of an arrow at the centre of vision allowed patients to scan and search with visual instructions. The cue added a bottom-up stimulus-based approach, which does not necessitate patient awareness of the deficit. The utility of this combination is supported by previous work describing the segregation of goal-directed (top-down) and stimulus-driven (bottom-up) attention into the dorsal and ventral frontoparietal cortices, respectively (Corbetta and Shulman, 2002). Moreover, the use of a centrally displayed arrow provides correction of the visuospatial standard reference frame because the central position presents more definite and precise coordinates, irrespective of head or eye location. Moreover, the immersive property from VR heightens attention, thus enhancing the therapeutic effect of HSN.

**FIGURE 6:**
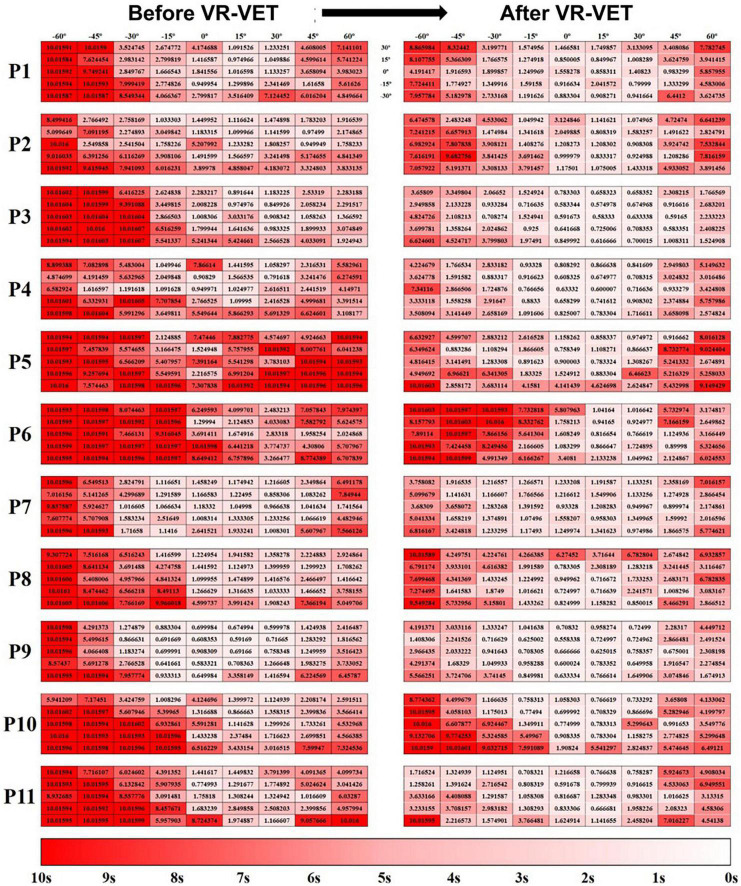
Hit-map of the field of regard (FOR) response times. **(Left panel)** Before VR-VET training and **(right panel)** after 20 sessions of VR-VET. Response times close to 0 s are shown in white, and those close to 10 s are shown in red. P, participant; VR-VET, virtual reality-based visual exploration therapy.

Additionally, this VR-based HSN assessment shows the changing pattern of HSN easily throughout the intervention without any separate evaluation. It gives more in-depth information about HSN than clinical assessment, including FOP and FOR, and information dividing space into either right or left hemispace, as demonstrated in a previous study (Kim et al., 2021). The present study also suggested that the same system provided more detailed pictures of the individualised neglect maps and therapeutic effects as shown in Figure 6. In addition, this detailed figure, although subjective, suggests that the VR-VET effects can range from mild to severe.

RehAtt™, which is made up of VR-based scanning training and haptic stimuli, has shown improvement of HSN (Fordell et al., 2016). Although it is difficult to differentiate the role of VR-based scanning training and haptic stimuli, the positive results from our study, which did not use haptic device, suggest that the VR-based exploration might play a role in HSN rehabilitation. In addition, we presented visual exploration training using HMD, whereas RehAtt™ presented virtual environment with CRT monitor and 3D vision glasses. Although the 3D vision glasses have their own advantages, including ease of use or affordability, the HMD enables more immersive virtual experience via high resolution display and further blocking the outer world, which is known to be linked to motivation, feeling of entertainment, and good compliance (Martino Cinnera et al., 2022). In addition, our VR-VET utilized a computer mouse, while RehAtt™ operated a robotic pen; hence, VR-VET could be easily adopted for in-home rehabilitation or telerehabilitation.

The present study, however, has few limitations. First, our results are preliminary and require validation in a randomised controlled trial comparing our intervention with other HSN interventions. Second, the sample size was small. We hypothesized that significant results could be easily obtained from a small sample size because the FOPR test enables highly sensitive quantification of visuospatial function, which is not achievable through conventional tests (Kim et al., 2021). However, a small number of participants in previous studies on HSN has been a major issue to establish evidence of an effective intervention method for HSN (Ogourtsova et al., 2017; Longley et al., 2021). Thus, further studies involving a greater number of patients are warranted to validate the effectiveness of our VR-VET. Third, the participants were heterogenous in terms of stroke onset. We performed experiments with within-subject control, in which only patients who did not show improvements were included in the study to differentiate the effects of VR-VET from those due to spontaneous recovery. A previous intervention adopting scanning, in a manner similar to our VR-VET protocol, demonstrated that the effects came from the intervention *per se*, ruling out the effects of spontaneous recovery (Pizzamiglio et al., 2006). However, the HSN severity or characteristics might differ according to the time after stroke onset, and the lack of marked change of the clinical assessment may be due to the large variation of the stroke onset duration; thus, our findings must be interpreted with various considerations (Karnath et al., 2011). Fourth, we did not utilize more detailed clinical HSN tests, such as entire BIT battery. These standardized tests, if used, would have helped further present more reliable and valid findings. Fifth, VR-VET presented the visual targets equally in the right and left hemispaces following previously developed FOPR test. It is interesting to compare VR-VET incorporating more left space-focused stimuli with the current protocol of VR-VET. In addition, personalised VR-VET incorporating real-time cue and stimuli based on the current performance are recommended, considering the heterogeneity of neglect with regard to spatial representation and patterns of recovery (Pierce and Buxbaum, 2002; Buxbaum et al., 2004; Dvorkin et al., 2012). Sixth, our VR program used an experimental virtual space. Future integration of real-world components into the program may allow for further improvements in real-world navigational behaviour.

## 5. Conclusion

Our findings suggest that our VR-VET improved HSN with beneficial effects on both visuospatial perceptual and exploratory function regarding whole space, especially left hemispace, representing multi-dimensional effects. Additionally, the effects were also observed in clinical assessments, indicating the translatability of these improvements to real-world function.

## Data availability statement

The raw data supporting the conclusions of this article are available from the corresponding author on reasonable request.

## Ethics statement

The studies involving human participants were reviewed and approved by the Institutional Review Board of National Rehabilitation Centre. The patients/participants provided their written informed consent to participate in this study.

## Author contributions

J-HS contributed to the conception and design of the VR training, analysis and interpretation of data, and drafting of the manuscript. MK provided the technical support, contributed to the analysis of data, and drafting of the manuscript. J-YL, M-YK, and Y-JJ involved in the clinical management, data collection, and interpretation of the results. KK provided the technical and material support, conception of the VR training, performed the statistical data analysis, interpreted the data, and critically reviewed the manuscript for important intellectual content. All authors read and approved the final manuscript.

## Footnotes

1
http://www.r-project.org
