# Supplementary material for: Feasibility of hemispatial neglect rehabilitation with virtual reality-based visual exploration therapy among patients with stroke: randomised controlled trial
Source: Front Neurosci. 2023 Apr 20;17:1142663. doi: 10.3389/fnins.2023.1142663 (PMC10157074; doi:10.3389/fnins.2023.1142663)
Supplement: Supplementary file 1 [file Data_Sheet_1.docx]

Supplementary Material

# Rehabilitation of Hemispatial Neglect with Virtual reality-based Visual Exploration Therapy among patients with stroke: Randomised Controlled Trial

**Joon-Ho Shin, Mingyu Kim, Ji-Yeong Lee, Mi-Young Kim, Yu-Jin Jeon, Kwanguk Kim^*^**

***Correspondence:** Dr. Kwanguk (Kenny) Kim, Email: [kenny@hanyang.ac.kr](mailto:kenny@hanyang.ac.kr)

# Supplementary Table Effects of VR-VET and WT on FOPR tests in near and far space

|  | VR group (n=11) | | | | | | WT group (n=11) | | | | | | Interaction |  | Main effect: Time | | Main effect: Type | |
| --- | --- | --- | --- | --- | --- | --- | --- | --- | --- | --- | --- | --- | --- | --- | --- | --- | --- | --- |
|  | Before | | | After | | | Before | | | After | | | F | P | F | P | F | P |
| FOP-NEAR-RT (sec) | 1.873 | ± | 0.721 | 1.415 | ± | 0.706 | 1.776 | ± | 0.825 | 1.529 | ± | 0.774 | 1.600 | 0.235 | 15.287 | 0.003 | 0.003 | 0.955 |
| FOP-NEAR-LEFT-RT (sec) | 3.037 | ± | 1.424 | 2.194 | ± | 1.365 | 2.598 | ± | 1.201 | 2.510 | ± | 1.567 | 7.334 | 0.022 | 10.600 | 0.009 | 0.092 | 0.768 |
| FOP-NEAR-RIGHT-RT (sec) | 1.056 | ± | 0.521 | 0.850 | ± | 0.261 | 1.222 | ± | 0.704 | 0.809 | ± | 0.191 | 1.154 | 0.308 | 7.186 | 0.023 | 0.175 | 0.685 |
| FOP-NEAR-SR (%) | 76.364 | ± | 17.667 | 87.879 | ± | 14.006 | 80.303 | ± | 20.136 | 83.939 | ± | 16.655 | 2.191 | 0.170 | 14.689 | 0.003 | 0.021 | 1.000 |
| FOP-NEAR-LEFT-SR (%) | 55.303 | ± | 34.414 | 73.485 | ± | 30.004 | 65.909 | ± | 30.381 | 65.152 | ± | 33.915 | 5.157 | 0.047 | 4.862 | 0.052 | 0.056 | 0.818 |
| FOP-NEAR-RIGHT-SR (%) | 91.667 | ± | 9.860 | 97.727 | ± | 5.389 | 90.152 | ± | 16.167 | 96.970 | ± | 5.618 | 0.028 | 0.871 | 6.784 | 0.026 | 0.127 | 0.728 |
|  |  |  |  |  |  |  |  |  |  |  |  |  |  |  |  |  |  |  |
| FOP-FAR-RT (sec) | 2.215 | ± | 0.706 | 1.629 | ± | 0.689 | 2.028 | ± | 0.859 | 1.699 | ± | 0.722 | 3.187 | 0.105 | 16.149 | 0.002 | 0.101 | 0.757 |
| FOP-FAR-LEFT-RT (sec) | 3.605 | ± | 1.058 | 2.627 | ± | 1.295 | 3.117 | ± | 1.220 | 2.764 | ± | 1.367 | 8.741 | 0.014 | 16.444 | 0.002 | 0.450 | 0.518 |
| FOP-FAR-RIGHT-RT (sec) | 1.157 | ± | 0.745 | 0.932 | ± | 0.328 | 1.308 | ± | 0.720 | 0.864 | ± | 0.179 | 0.974 | 0.347 | 6.182 | 0.032 | 0.058 | 0.814 |
| FOP-FAR-SR (%) | 69.697 | ± | 17.092 | 83.030 | ± | 15.308 | 74.545 | ± | 22.621 | 80.000 | ± | 16.733 | 2.493 | 0.145 | 15.158 | 0.003 | 0.057 | 0.817 |
| FOP-FAR-LEFT-SR (%) | 39.394 | ± | 29.129 | 63.636 | ± | 28.204 | 53.030 | ± | 32.333 | 57.576 | ± | 32.157 | 6.940 | 0.025 | 10.359 | 0.009 | 0.365 | 0.559 |
| FOP-FAR-RIGHT-SR (%) | 92.424 | ± | 15.117 | 96.212 | ± | 6.835 | 87.879 | ± | 18.769 | 97.727 | ± | 3.892 | 0.558 | 0.472 | 6.612 | 0.028 | 0.201 | 0.664 |
|  |  |  |  |  |  |  |  |  |  |  |  |  |  |  |  |  |  |  |
| FOR-NEAR-RT (sec) | 4.621 | ± | 1.430 | 2.823 | ± | 1.025 | 3.849 | ± | 1.457 | 3.856 | ± | 1.340 | 11.797 | 0.006 | 31.162 | 0.000 | 0.163 | 0.695 |
| FOR-NEAR-LEFT-RT (sec) | 6.841 | ± | 1.776 | 3.871 | ± | 1.876 | 5.118 | ± | 2.139 | 5.600 | ± | 2.327 | 17.876 | 0.002 | 41.936 | 0.000 | 0.000 | 0.900 |
| FOR-NEAR-RIGHT-RT (sec) | 2.983 | ± | 1.723 | 2.203 | ± | 0.713 | 3.063 | ± | 1.765 | 2.612 | ± | 0.983 | 0.221 | 0.648 | 4.477 | 0.060 | 0.512 | 0.491 |
| FOR-NEAR-SR (%) | 72.929 | ± | 15.000 | 91.919 | ± | 10.197 | 81.212 | ± | 15.756 | 83.636 | ± | 16.489 | 8.625 | 0.015 | 24.880 | 0.001 | 0.000 | 1.000 |
| FOR-NEAR-LEFT-SR (%) | 48.636 | ± | 24.504 | 85.455 | ± | 20.791 | 70.000 | ± | 28.373 | 66.364 | ± | 32.718 | 19.519 | 0.001 | 30.947 | 0.000 | 0.036 | 0.854 |
| FOR-NEAR-RIGHT-SR (%) | 91.818 | ± | 17.503 | 96.818 | ± | 4.045 | 90.000 | ± | 17.321 | 98.182 | ± | 3.371 | 0.187 | 0.675 | 3.775 | 0.081 | 0.003 | 0.956 |
|  |  |  |  |  |  |  |  |  |  |  |  |  |  |  |  |  |  |  |
| FOR-FAR-RT (sec) | 5.350 | ± | 1.483 | 3.217 | ± | 1.162 | 4.541 | ± | 1.677 | 4.472 | ± | 1.502 | 15.600 | 0.003 | 31.125 | 0.000 | 0.320 | 0.584 |
| FOR-FAR-LEFT-RT (sec) | 7.302 | ± | 1.611 | 4.028 | ± | 1.963 | 5.695 | ± | 2.415 | 6.070 | ± | 2.376 | 24.279 | 0.001 | 41.555 | 0.000 | 0.185 | 0.677 |
| FOR-FAR-RIGHT-RT (sec) | 3.813 | ± | 1.876 | 2.826 | ± | 0.969 | 3.910 | ± | 1.853 | 3.281 | ± | 1.168 | 0.240 | 0.635 | 7.536 | 0.021 | 0.527 | 0.484 |
| FOR-FAR-SR (%) | 65.253 | ± | 17.951 | 90.707 | ± | 10.319 | 75.354 | ± | 21.761 | 75.758 | ± | 17.426 | 12.752 | 0.005 | 33.809 | 0.000 | 0.279 | 0.609 |
| FOR-FAR-LEFT-SR (%) | 43.182 | ± | 22.279 | 85.000 | ± | 20.248 | 65.000 | ± | 30.984 | 59.091 | ± | 31.210 | 25.917 | 0.000 | 49.928 | 0.000 | 0.109 | 0.748 |
| FOR-FAR-RIGHT-SR (%) | 83.636 | ± | 22.259 | 94.545 | ± | 4.719 | 81.364 | ± | 22.259 | 89.545 | ± | 10.357 | 0.069 | 0.798 | 8.927 | 0.014 | 0.735 | 0.411 |

# Supplementary Figures


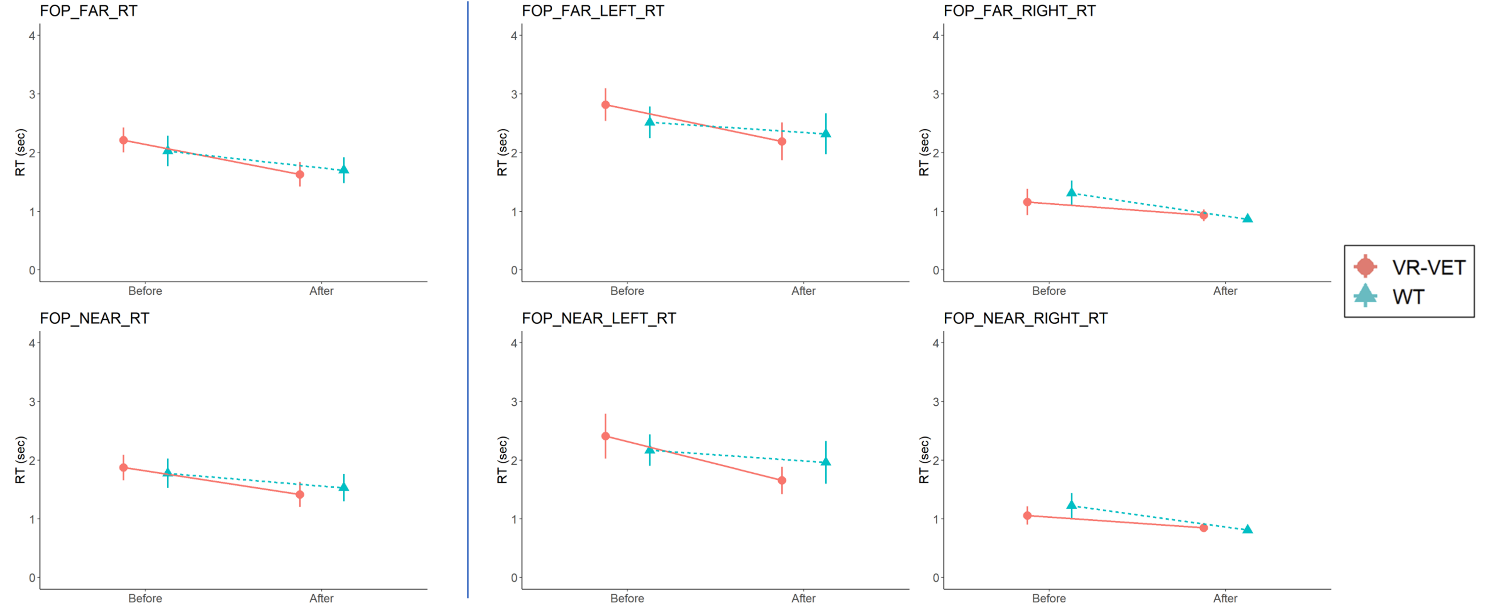


**Supplementary Figure 1.** Comparisons of FOP-RT in left space between VR-VET and waiting in far and near space.

FOP, field of perception; RT, response time; VR-VET, virtual reality-visual exploration therapy.


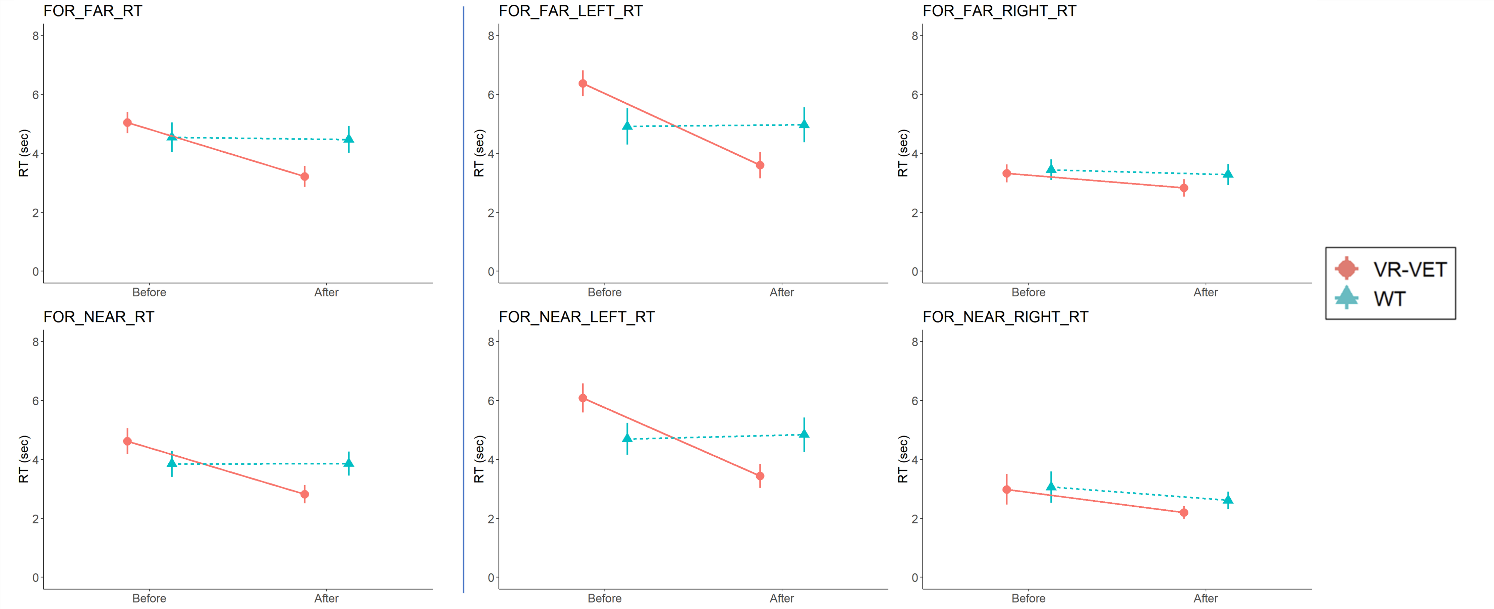


**Supplementary Figure 2.** Comparisons of FOR-RT in left space between VR-VET and waiting in far and near space.

FOR: field of regard; RT, response time; VR-VET, virtual reality-visual exploration therapy.


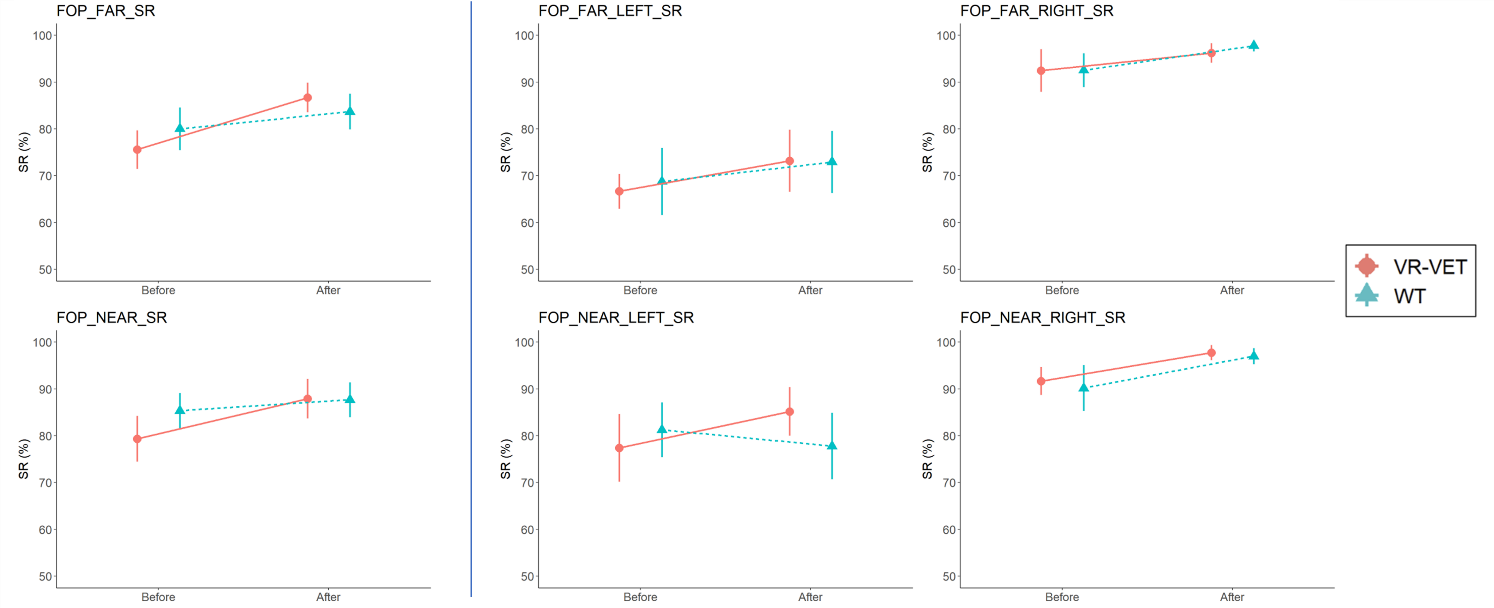


**Supplementary Figure 3.** Comparisons of FOP-SR in left space between VR-VET and waiting in far and near space.

FOP: field of perception; SR, success rate; VR-VET, virtual reality-visual exploration therapy.


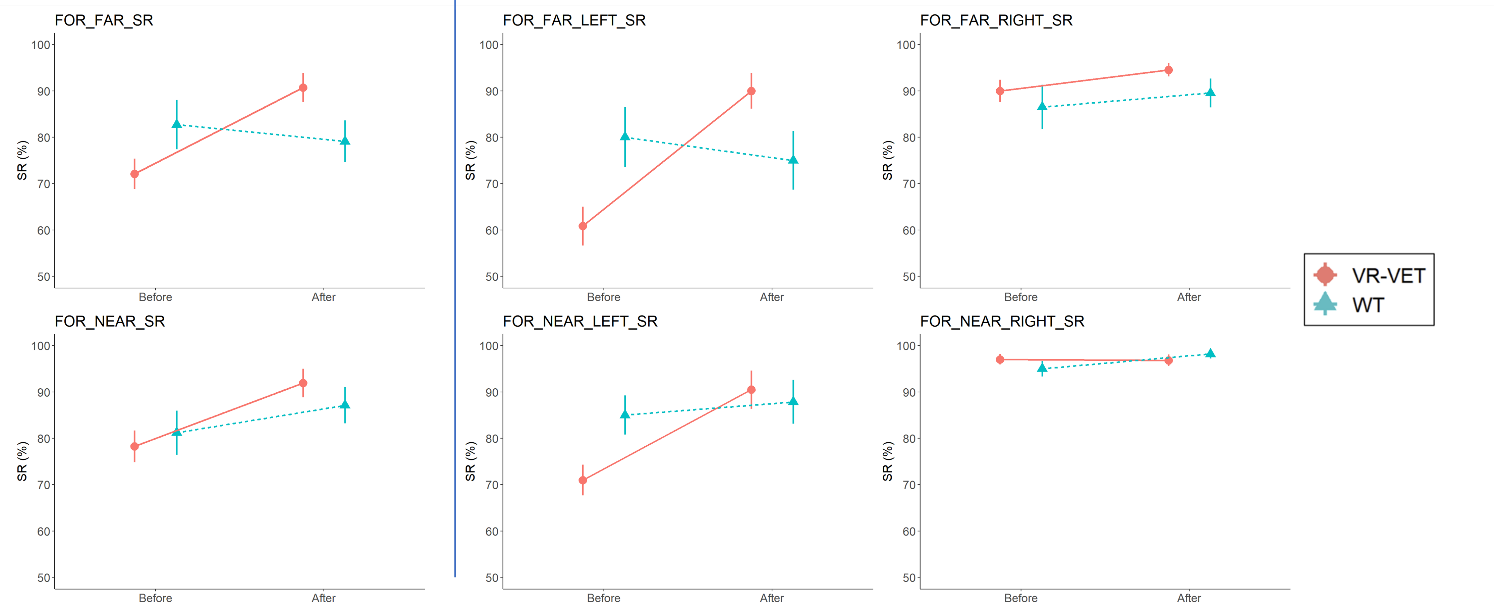


**Supplementary Figure 4.** Comparisons of FOR-SR in left space between VR-VET and waiting in far and near space.

FOR: field of regard; SR, success rate; VR-VET, virtual reality-visual exploration therapy.
